# Differential Effects of Bisphenol A and Diethylstilbestrol on Human, Rat and Mouse Fetal Leydig Cell Function

**DOI:** 10.1371/journal.pone.0051579

**Published:** 2012-12-17

**Authors:** Thierry N’Tumba-Byn, Delphine Moison, Marlène Lacroix, Charlotte Lecureuil, Laëtitia Lesage, Sophie M. Prud’homme, Stéphanie Pozzi-Gaudin, René Frydman, Alexandra Benachi, Gabriel Livera, Virginie Rouiller-Fabre, René Habert

**Affiliations:** 1 Université Paris Diderot, Sorbonne Paris Cité, Laboratory of Development of the Gonads, Unit of Stem Cells and Radiation, Fontenay-aux-Roses, France; 2 CEA, DSV, iRCM, SCSR, LDG, Fontenay-aux-Roses, France; 3 INSERM, Unité 967, Fontenay aux Roses, France; 4 INRA, UMR1331, TOXALIM, Toulouse, France; 5 Service de Gynécologie-Obstétrique et Médecine de la Reproduction, Hôpital A. Béclère, Université Paris Sud, Clamart, France; Clermont-Ferrand Univ., France

## Abstract

Endocrine disruptors (ED) have been incriminated in the current increase of male reproductive alterations. Bisphenol A (BPA) is a widely used weak estrogenic environmental ED and it is debated whether BPA concentrations within the average internal exposure are toxic. In the present study we investigated the effects of 10^−12^ to 10^−5^ M BPA concentrations on fetal Leydig cell function, as fetal life is a critical period of sensitivity to ED effects on male reproductive function. To this aim, fetal testes from human at 6.5–10.5 gestational weeks (GW) or from rat and mouse at a comparable critical period of development (14.5 days post-coitum (dpc) for rat and 12.5 dpc for mouse) were explanted and cultured using our validated organotypic culture system in the presence or absence of BPA for 1–3 days. BPA concentrations as low as 10^−8^ M reduced testosterone secretion by human testes from day 1 of culture onwards, but not by mouse and rat testes where concentrations equal to 10^−5^ M BPA were required. Similarly, 10^−8^ M BPA reduced *INSL3* mRNA levels only in human cultured testes. On the contrary, 10^−5^ and 10^−6^ M diethylstilbestrol (DES), a classical estrogenic compound, affected testosterone secretion only in rat and mouse testis cultures, but not in human testis cultures. Lastly, contrarily to the DES effect, the negative effect of BPA on testosterone produced by the mouse fetal testis was maintained after invalidation of estrogen receptor α (ERα). In conclusion, these results evidenced i) a deleterious effect of BPA on fetal Leydig cells function in human for concentrations from 10^−8^ M upwards, ii) species-specific differences raising concerns about extrapolation of data from rodent studies to human risk assessment, iii) a specific signaling pathway for BPA which differs from the DES one and which does not involve ERα.

## Introduction

Concerns about the increasing incidence of abnormalities in human and animal male reproductive function, such as cryptorchidism, hypospadias, low sperm count and testicular germ cell cancer, have been steadily increasing [1], [2]. These disorders have been hypothesized to be the expression of one common underlying disorder, the testicular dysgenesis syndrome (TDS) that arises during fetal life [3], [4]. Specifically, the higher occurrence of cryptorchidism and hypospadias might be the result of increasing alterations of the function of fetal Leydig cells. Indeed, Leydig cells produce testosterone that is responsible for the masculinization of the male urogenital system and external genitalia [5]–[8]. Moreover, fetal testis migration into the scrotum is dependent on testosterone and Insulin-like 3 (INSL3), a hormone produced by Leydig cells [9], [10]. An important finding in relation to TDS is that androgen action during the “masculinization programming window”, which extends from 15.5 days post-conception (dpc) to 18.5 dpc in the rat, (equivalent of 13 to 17.5 dpc in the mouse and estimated to be from 6.5 to 14 gestational week (GW) in human) is essential for normal phenotypic masculinization [11], [12].

Since the initial “estrogenic hypothesis” proposed by Sharpe and Skakkebaek, several findings have suggested a link between deterioration of reproductive health and environmental factors, particularly endocrine disruptors (EDs), that have been quantitatively and qualitatively increasing in our environment during the last decades [3], [6], [7], [13].

Among such EDs, the estrogenic activity of bisphenol A (BPA, 4,4′-dihydroxy-2,2-diphenylpropane) has been the focus of considerable research effort and discussion about its toxicity at low doses [14]–[17]. BPA is a widely used and produced organic compound (about 3.5 million tons manufactured worldwide in 2008). Currently, it is used as monomer for the industrial production by polymerization of polycarbonate plastic (72%) and epoxy resins (21%), and as anti-oxidant or inhibitor of polymerization in some plasticizers and PVC (7%) [18]. BPA can leach into the content of food containers made of polycarbonate plastic or coated with epoxy resins and it is then ingested [18]. This is the main source of contamination, although its ubiquitous distribution leads also to contamination from dermal exposure and inhalation of household dusts.

Data on the effect of BPA on fetal Leydig cell function are limited. Exposure to high doses of BPA during pregnancy reduced plasma testosterone at birth and increased expression of Raf1, which is predominant in Leydig cells, at postnatal day 3 in the rat [19], [20]. Administration of doses 5 times higher than the recommended tolerable daily intake (*i.e*., 50 µg/kg/day) to pregnant rats reduced the anogenital distance in male pups, whereas lower BPA doses did not have any effect [21]. On the contrary, three other independent studies didn’t show any effect of BPA on AGD after a gestational gavage with 2–200 µg/kg/day, 4000–40000 µg/kg/day and 1–50000 µg/kg/day respectively [22]–[24]. In humans, a recent retrospective epidemiological study highlighted that sons of workers who were professionally exposed to high levels of BPA during pregnancy had shorter AGD [25]. However, no increase in BPA concentration in umbilical cord blood was observed in newborns with cryptorchidism [26].

In the 1990s, we developed an organotypic culture system for fetal testis from rats on floating filters in which the testis architecture and intercellular communications are preserved enough to allow reproducing the normal development of testis somatic and germ cells *in vitro* without addition of exogenous signaling factors [27]–[30]. We have then extended this methodology to the culture of mouse and human fetal testes [31], [32]. We proposed this organotypic culture system as a method to study the direct effects of potential EDs on fetal testicular functions and development under the name of rat, mouse and human Fetal Testis Assay (r/m/h FeTA) [6], [31], [33]. This experimental approach has already been employed to evaluate the effect of different potential EDs, such as DES [34], [35], mono-(2-ethylhexyl) phthalate (MEHP) [36]–[39], cadmium [40], mild analgesics [41] and genistein [42] on the fetal development of different cell types in both rodent and human testes. Recently, we further validated this organotypic culture system as a highly valuable model for toxicological assay by showing that it reproduces the *in vivo* observed effects of one phthalate on the mouse fetal testis development [39].

Using FeTA, here we evaluated the effect of different concentrations of BPA on human Leydig cell endocrine functions through quantification of testosterone secretion in the culture medium and of *INSL3* mRNA levels in the testis. We then compared these effects with those observed in rat and mouse fetal testes, which are classically used in toxicological studies. Finally we compared the BPA effects with those of diethylstilbestrol (DES), an estrogenic compound that is used as a positive control for BPA, in the three species and also in mouse invalidated for Estrogen Receptor α (ERα) to investigate the role of this receptor in BPA-signaling.

## Materials and Methods

### Animals

Wistar rats from Janvier (Le Genest Saint Isle, France) and NMRI and C57BL/6 mice bred in our animal facility were housed under controlled photoperiod conditions (lights from 08∶00 to 20∶00 h) and were supplied with estrogen-free commercial food and tap water ad libitum.

C57BL/6 mice heterozygous for ERα (ERα+/−) were produced by Dupont et al. [43] and generously provided by Prof. P. Chambon (IGBMC, Illkirch, France). Exon 3 of this gene, encoding the first zinc finger of the DNA-binding domain, was targeted for disruption. We generated mice homozygous for ERα (ERα−/−) by caging heterozygous males with heterozygous females.

The day after overnight mating was counted as 0.5 day post-conception (dpc). Pregnant mice were killed by cervical dislocation at 12.5 dpc and pregnant rats were anesthetized by intraperitoneal injection of 4 mg/ml sodium pentobarbital (Sanofi, Libourne, France) at 14.5 dpc. Fetuses were quickly removed from the uterus, dissected under a binocular microscope and sexed based on the gonad morphology as previously described [31]. Testes were removed without the adjacent mesonephros.

The animal facility is licensed by the French Ministry of Agriculture (agreement N°B92-032-02). All animal experiments were supervised by Pr. René Habert (agreement delivered by the French Ministry of Agriculture for animal experiment N°92–191) in compliance with the NIH Guide for Care and Use of Laboratory Animals. All efforts were made to minimize animal suffering.

### Collection of Human Fetal Testes

Human fetal testes from the 6^th^ until the 11^th^ GW were obtained from pregnant women referred to the Department of Obstetrics and Gynecology at the Antoine Béclère Hospital, Clamart (France) for legally induced abortions in the first trimester of pregnancy as previously described [32]. All pregnant women provided their written informed consent for scientific use of the fetuses. None of the terminations were due to fetal abnormality. Fetuses were dissected under a binocular microscope. The fetal age was evaluated by measuring the length of limbs and feet [44]. Gonads within the abortive material were retrieved in approximately 50% of cases. Testes were identified based on their morphology. The project was approved by the local Medical Ethics Committee and by the French Biomedecine Agency (reference number PFS12-002).

### Organ Cultures

The organotypic culture system was previously described [27], [31], [32]. Briefly, human, rat and mouse testes were cultured on Millicell-CM Biopore membranes (pore size 0.4 µm, Millipore, Billerica, MA) floating on 320 µL culture medium in tissue culture dishes at 37°C in a humidified atmosphere containing 95% air/5% CO_2_. The culture medium was phenol-red free Dulbecco modified Eagle medium/Ham F12 (1∶1) (Gibco, Grand Island, NY), supplemented with 80 µg/mL gentamicin (Sigma, St. Louis, MO). BPA and DES were purchased from Sigma (Sigma, St. Louis, MO) and diluted in ethanol. The whole culture medium was changed every 24 h.

For mouse and rat, one entire testis was placed on the membrane. For human, each testis was cut into small pieces due to their larger size (12 to 36 pieces depending on the age of the testis) and 3–4 pieces were randomly placed on the membrane providing 4 to 8 wells per testis. Thus, the size of the explants was comparable for the 3 species.

For evaluating the effects of BPA or DES on testosterone secretion, in human one testis per fetus was used. Explants were first cultured in control culture medium for 24 h (D0). Then the culture were pursued for 3 days (D1 to D3) with half of the wells added with BPA or DES and the other half in basal medium to serve as controls. For each well, the D1 to D3 testosterone secretion in the medium was normalized to its basal secretion at D0. Then, the average of the normalized secretion of the treated explants was divided by that of untreated explants to obtain the “relative secretion”. This protocol has been previously validated [32], [37], [40]. For rat and mouse, the protocol was identical except that for each fetus, one entire testis was treated with BPA or DES, whereas the other one was left untreated and served as control.

For assessing the effect of BPA on *INSL3* expression, one testis (mouse and rat) or the pool of all the pieces from one testis (human) cultured in the presence of BPA was compared with the untreated contralateral testis (mouse and rat) or the pool of all the pieces from the untreated contralateral testis (human).

As BPA in the culture dishes can leach in the medium [45], we measured the BPA concentration in the medium before and after culture by using the LC/MS method developed for biological fluids [46]. Culture medium samples were directly assayed after dilution (1∶1) with the internal standard solution (200 ng/mL BPA-d-16 in AcN). The limit of sensitivity was 1 ng/ml (4.38.10^−9^ M BPA). No BPA was detected in control samples (no BPA added) before and after culture, whereas the expected concentrations of BPA were detected in treated samples.

### Histology

At the end of the culture some explants were fixed for 2 h at 4°C in Bouin’s fluid. After dehydratation in absolute ethanol and 1-butanol the tissue pieces were embedded in paraffin wax and sectioned at 5 µm thickness. The sections were mounted on slides, deparaffined, rehydrated and stained with hematoxylin as previously described [28].

### Testosterone Radioimmunoassay

We measured the amount of testosterone secreted into the culture medium every 24 hours by radioimmunoassay as previously described [47]. The limit of detection was 8 pg/100 µl. The intra-assay coefficient of variation (CoV) is 2% and inter-assay CoV is 5%.

### RNA Extraction, Reverse Transcription and Real-Time Polymerase Chain Reaction

Explants were frozen in RLT buffer (Qiagen, Courtaboeuf, France) at the end of the culture period and stored at −20°C. Total RNA from frozen samples was extracted using the RNeasy Plus Mini-Kit (Qiagen, Courtaboeuf, France) and reverse transcribed using the High Capacity cDNA Reverse Transcription Kit (Applied Biosystems, Courtabeuf, France), followed by real-time PCR as previously described [32]. Primers and probes were designed by Applied Biosystems (sequences not provided: human *β-Actin*, NM_001101.2; mouse *β-Actin,* NM_007393.3; rat *β-Actin,* NM_031144.2; human *INSL3*, Hs01394273_m1; mouse *Insl3*, Mm01340353_m1; rat *Insl3*, Rn00586632_m1). Reactions were carried out and fluorescence detected using an ABI Prism 7900 apparatus (Applied Biosystems, Courtabeuf, France). Each sample was run in duplicate. Negative controls were run for every primer/probe combination. The measured amount of cDNA for *Insl3* was normalized to *ß-Actin*.

### Statistical Analysis

All values are expressed as mean ± SEM. The significance of the differences between mean values of treated and untreated explants was evaluated using the Wilcoxon’s non-parametric paired test. The statistical comparison between basal testosterone secretion values from ERα+/+, ERα+/−, ERα−/− animals was performed with the Mann-Whitney’s non-parametric unpaired test.

## Results

### Effect of BPA on Fetal Testis Morphology

**Figure 1:**
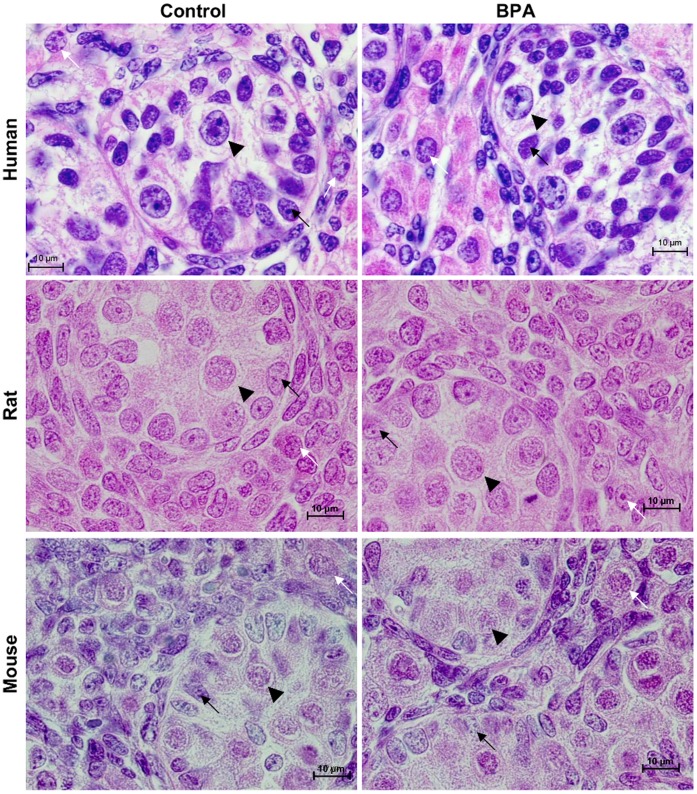
Effect of BPA treatment on testicular histology. Histological sections of human, rat and mouse fetal testes removed at 11 gestational week (GW), 14.5 day post conception (dpc) and 12.5 dpc respectively after one day of culture in control medium (D0) followed by 3 days of culture in the absence (control) or presence (BPA) of 10^−5^ M BPA. At the end of the culture, testes were fixed in Bouin’s fluid and hematoxylin/eosin staining of the histological sections was performed. The testicular architecture and morphology were not affected by BPA-treatment. Black arrows: Sertoli cells; white arrows: Leydig cells; arrowhead: gonocytes.

After culture in presence of 10^−5^ M BPA, the architecture of the testis was well maintained (Fig. 1). The testicular morphology, with the Sertoli cells surrounding the germ cells in the seminiferous cords and the Leydig cells in the interstitial compartment, was not altered by BPA treatment. We observed no sign of necrosis or disorganized areas in the tissue.

### Effect of BPA on Human Fetal Testis Steroidogenesis

**Figure 2:**
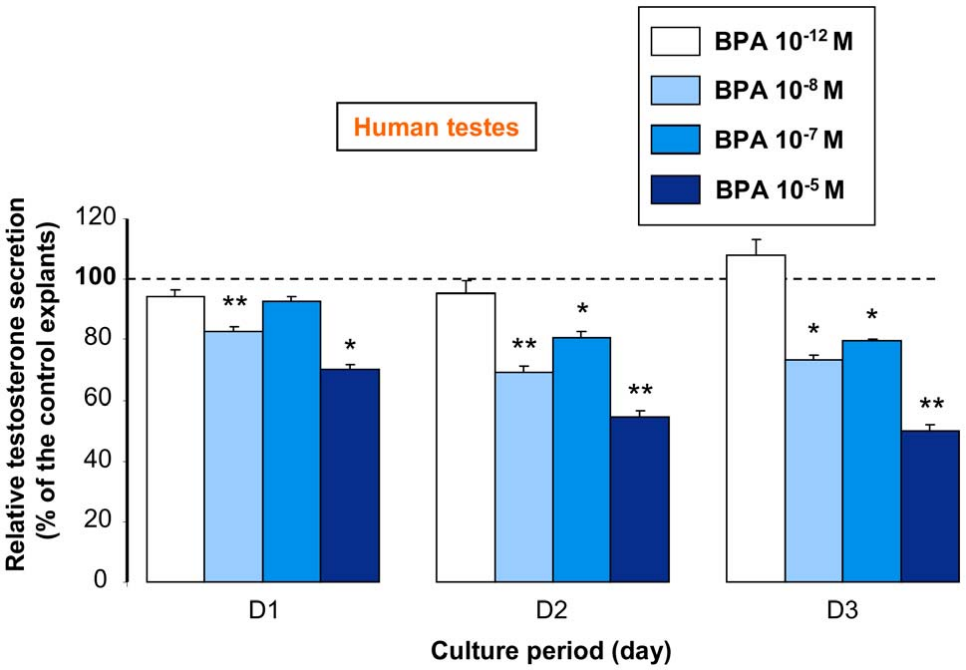
Effect of BPA on testosterone secretion by human fetal testes. One testis per fetus (between 6.5 and 10.5 gestational week) was cut in different pieces that were cultured as separated explants in different wells (4 to 12 wells according to the age of the fetus). After 24 h of culture in control medium (D0), half of the explants were cultured in the absence (control) and the other half in the presence of BPA at concentrations ranging from 10^−12^ M to 10^−5^ M for 3 days (D1 to D3). The daily testosterone secretion in treated and untreated samples was measured by radioimmunoassay and the values at D1 to D3 were normalized to the D0 secretion of the same well. The D1 to D3 mean normalized secretion of treated samples are expressed as the percentage of that of the controls (untreated samples). Means ± SEM of these percentages from n fetuses are presented. n = 5 for 10^−12^ M BPA, n = 7−8 for the other BPA concentrations. *p<0.05, ** p<0.01 in the statistical comparison between BPA-treated and control testes using the the Wilcoxon’s non-parametric paired test.

To investigate whether low doses of BPA could affect the activity of human fetal Leydig cells, organotypic cultures of human fetal (6.5–10.5 gestational weeks (GW)) testes were exposed to various BPA concentrations (from 10^−12^ to 10^−5^ M) for three days and testosterone secretion in the medium was quantified. Testosterone secretion was unaffected by 10^−12^ M BPA. Conversely, a BPA concentration as low as 10^−8^ M was enough to significantly decrease by 20% testosterone secretion during the first day of exposure (D1), with a maximum effect from D2 onwards (−30%) (Fig. 2).

**Figure 3:**
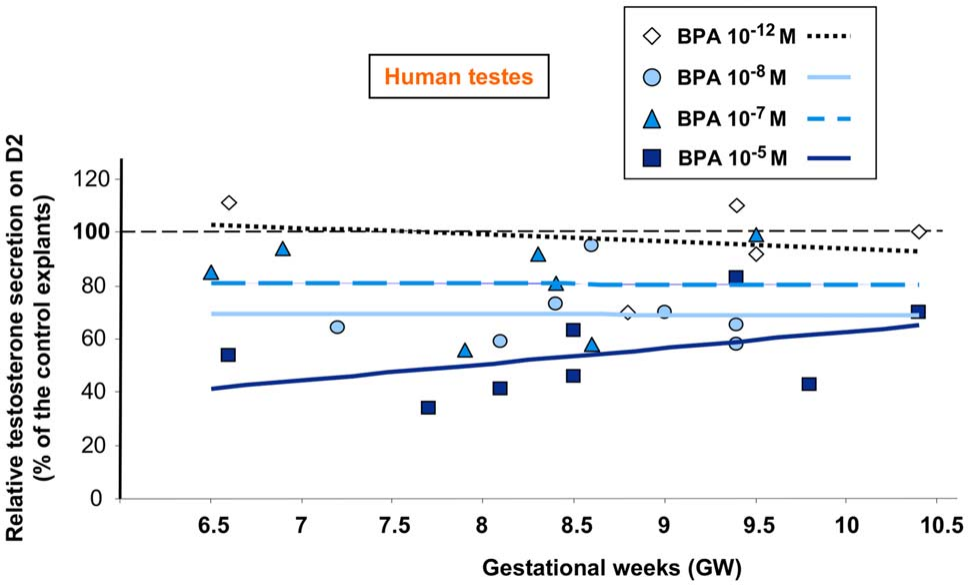
Effect of BPA on testosterone secretion by human fetal testes as a function of their developmental stage. Individual (normalized to D0 and expressed as the percentage of the control explants) values at D2 of culture for the samples of the experiment described in Figure 2 are presented. GW: gestational week.

The amount of testosterone secreted in the medium was largely dependent on the developmental stage as previously reported [32]. Specifically, at D0 (before addition of BPA), testes explanted at 6.5–7.4, 7.5–8.4, 8.5–9.4 and 9.5–10.5 GW secreted 252±83 pg/h (n = 5), 2721±1016 pg/h (n = 7), 5363±1038 pg/h (n = 10) and 13879±4231 pg/h (n = 5) of testosterone, respectively. Conversely, the effect of BPA on testosterone secretion was not influenced by the age of the fetus (Fig. 3).

### Effect of BPA on Rat and Mouse Fetal Testis Steroidogenesis

**Figure 4:**
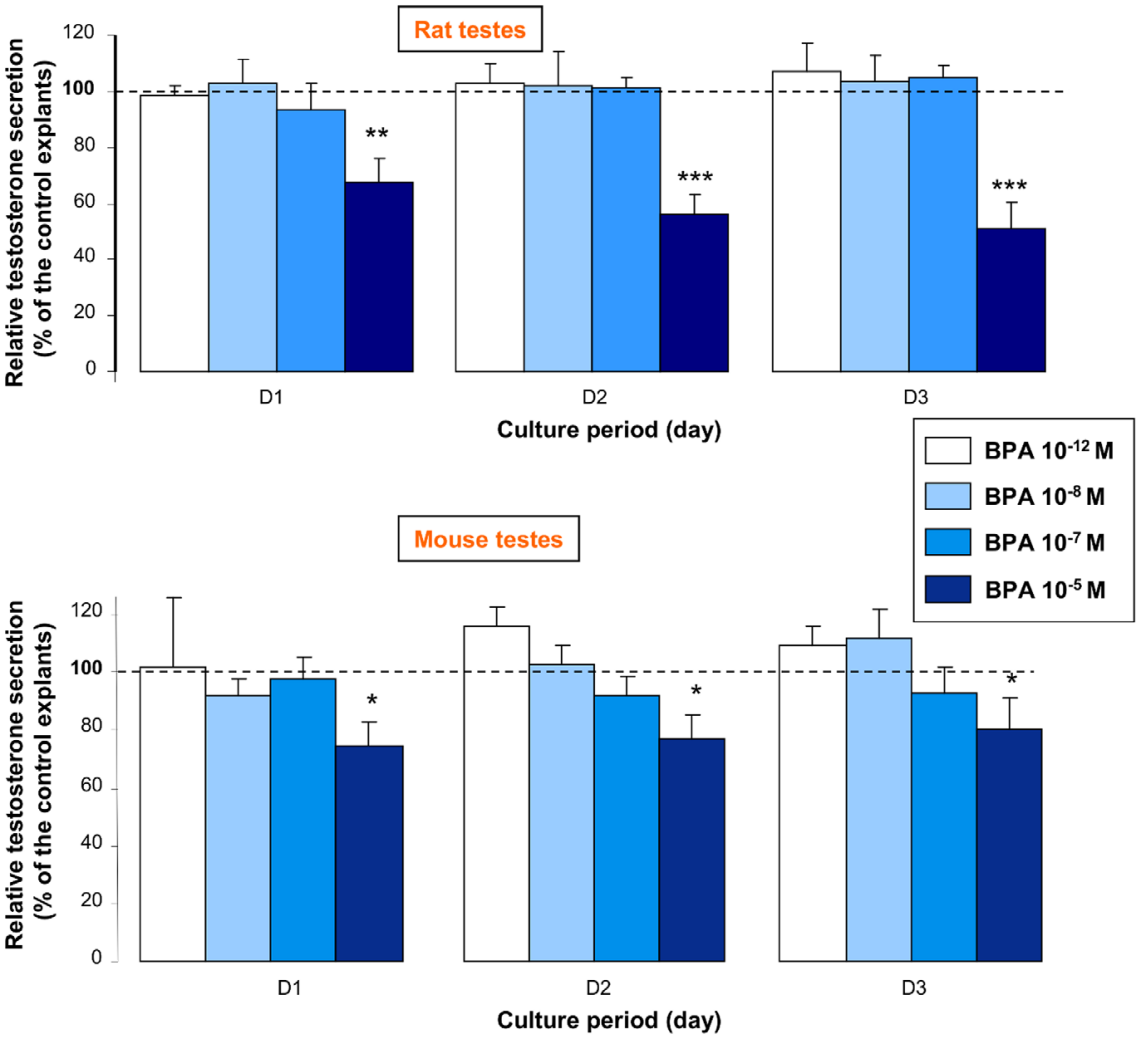
Effect of BPA on testosterone secretion by rat and mouse fetal testes. Testes were removed from 14.5 dpc rat and 12.5 dpc mouse fetuses and cultured for one day in control medium (D0). Then, for each fetus, one testis was kept in control medium and the other one in medium supplemented with various concentrations of BPA as indicated for 3 days (D1 to D3). Values were calculated and expressed as in Figure 1; Rat: n = 9 for 10^−12^ M, n = 12 for 10^−8^ M, n = 9 for 10^−7^ M, and n = 7 for 10^−5^ M. Mouse: n = 9 for 10^−12^ M, n = 15 for 10^−8^ M, n = 17 for 10^−7^ M and n = 10 for 10^−5^ M. *p<0.05, ** p<0.01, *** p<0.001 in the statistical comparison between BPA-treated and control testes using the Wilcoxon’s non-parametric paired test.

The same experiment was then carried out using testes from rat and NMRI mouse fetuses at a developmental stage that was comparable to that of the human testes (i.e., 14.5 for rat and 12.5 dpc for mouse). Differently from human testes, only the highest BPA concentration tested (10^−5^ M) altered testosterone production in the rat and mouse testes, while concentrations equal to or lower than 10^−7^ M had no effect even after 3 days of treatment (Fig. 4). Indeed, in rat, testosterone secretion rate was 95±15 pg/testis/h at D2 of treatment with 10^−7^ M BPA and 104±8 pg/testis/h in control cultures (untreated testes) (n = 9). In mouse, the testosterone secretion rate was 53±9 pg/testis/h at D2 of treatment with 10^−7^ M BPA and 49±11 pg/testis/h in control cultures (untreated testes) (n = 6).

These results indicate that the steroidogenic function of fetal testis is more sensitive to BPA in humans than in rodents.

### Effect of BPA on INSL3 mRNA Level

**Figure 5:**
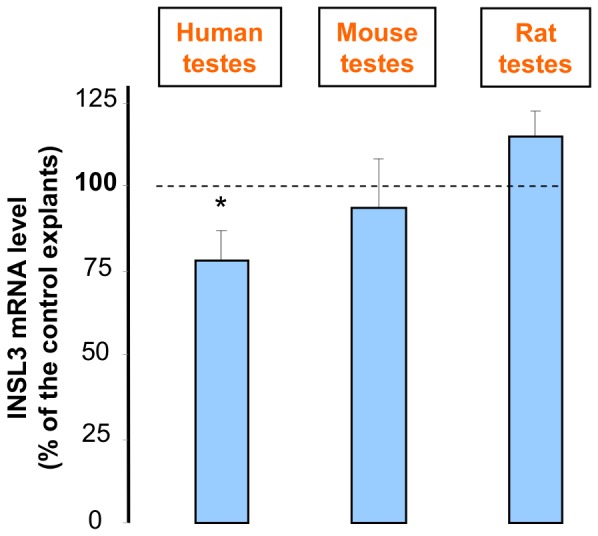
Effect of 10^−8^ M BPA on *INSL3* mRNA level in human, mouse and rat fetal testes. Testes were removed from human fetuses at 6.5 to 10.5 weeks of gestation or from mouse and rat fetuses at 12.5 and 14.5 dpc, respectively. For each fetus, both testes were cultured in control medium for 24 h (D0) and then one in the presence of 10^−8^ M BPA and the other one without for another 24 h. At the end of the culture, RT-PCR amplification was performed. Values are the mean ± SEM of *INSL3* mRNA level normalized to β-Actin one, and expressed as percentage of control values from 7 (human), 5 (mouse) and 6 (rat) independent experiments. * p<0.05 in the statistical comparison between BPA-treated and control testes using the paired Wilcoxon’s non-parametric paired test.

For each human, rat and NMRI mouse fetus, one testis was cultured in the presence of 10^−8^ M BPA for 24 h and the contralateral in its absence (control). This treatment reduced *INSL3* mRNA level only in human testes (by more than 20%), but not in rat and mouse testes (Fig. 5). Because, there was no effect in mouse and rat, we tested the effect of a higher concentration of BPA in the mouse. We observed that 10^−5^ M BPA induced a significant decrease in the *Insl3* mRNA level by around 20% (data not shown).

### DES Effect on Fetal Testis Steroidogenesis

**Figure 6:**
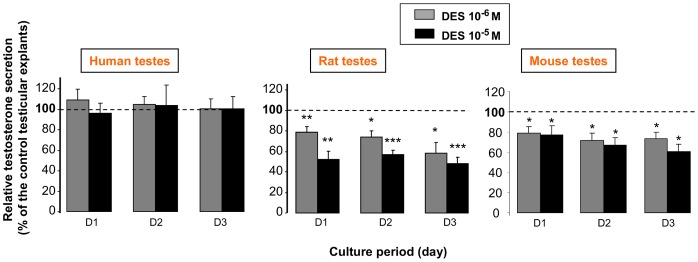
Effect of DES on testosterone secretion by human, rat and mouse fetal testes. Testes were removed from human fetuses at 6.5 to 10.5 weeks of gestation or from mouse and rat fetuses at 12.5 and 14.5 dpc, respectively. Like for the experiments presented in Figures 1 to 3, human rat and mouse testis explants were cultured in control medium (D0) for 24 h and then in the absence or presence of DES at 10^−6^ M or 10^−5^ M for the three subsequent days (D1–D3). Testosterone secretion in both treated and untreated samples at D1 to D3 was normalized to the D0 secretion of the same sample. The figure presents mean ± SEM of the D1 to D3 normalized values of treated samples expressed as the percentage of the control (untreated samples) values; n = 6, n = 7–10 and n = 7 for human, rat and mouse testes respectively. *p<0.05, ** p<0.01, *** p<0.001 in the statistical comparison between DES-treated and control testes using the Wilcoxon’s non-parametric paired test.

DES is commonly used as a positive control when assessing the estrogenic effects of EDs, particularly BPA. Surprisingly, addition of 10^−5^ or 10^−6^ M DES did not significantly modify testosterone production by human fetal testes in culture (Fig. 6). Conversely, testosterone secretion by 14.5 dpc rat fetal testes and by 12.5 dpc mouse testis was significantly reduced by addition of 10^−5^ M and 10^−6^ M DES (Fig. 6) as we previously reported using cultured fetal rat (14.5 dpc) and mouse (13.5 dpc) testes [34], [48].

### Effect of BPA on Mouse Fetal Steroidogenesis with Invalidation of ERα

We have previously studied the importance of ERα signaling in the effect of DES on mouse fetal steroidogenesis by comparing the amounts of testosterone secreted during 2 days by testes issued from ERα−/−, ERα+/− and ERα+/+ C57BL/6 mouse fetuses and cultured in the presence or absence of DES [48]. We reported that the negative effect of DES was abolished after invalidation of ERα. We used here the same mice and the same protocol (*i.e.* without D0 preculture) to investigate the ERα involvement in the effect of BPA.

First, as previously reported [48], we observed that the testosterone production in basal medium was much higher with ERα−/− animals (87.5 pg/testis/h, n = 5) than with wild-type animals (21.4 pg/testis/h, n = 6) (p<0.01 using Mann-Whitney’s non-parametric unpaired test). ERα+/− animals, that have only half the amount of ERα protein found in wild-type animal, produced amounts of testosterone intermediate between wild type and ERα−/− (38.3 pg/testis/h, n = 13) (p<0.01 in comparison with ERα−/− and ERα+/+ using Mann-Whitney’s non-parametric unpaired test). These results confirm that the estrogens endogenously produced by the cultured fetal testis inhibit testosterone production via ERα. Interestingly these results are positive controls in this experimental series for assessing the effects of BPA.

**Figure 7:**
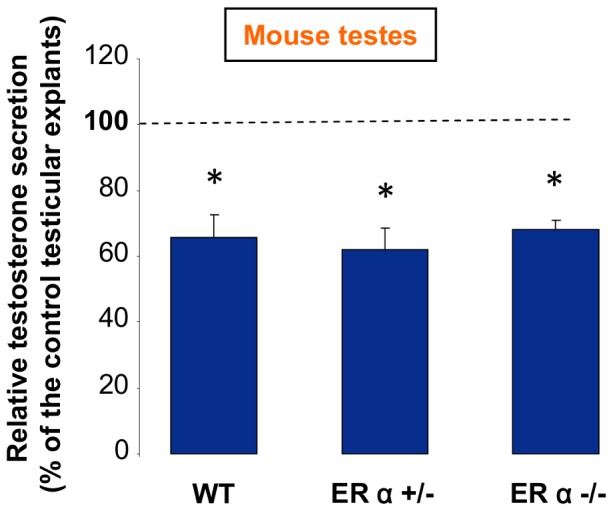
Effect of ERα gene inactivation on *in vitro* testicular response to BPA. Testes from homozygous 12.5 dpc (ERα−/−), heterozygous (ERα+/−) and wild-type (ERα+/+) ERα-deficient fetuses were cultured on floating filters for 48 h. One testis from each animal was cultured in control medium and the other one in medium containing 10^−5^ M BPA. Values are means ± SEM of testosterone secreted in the medium during the 2 days of culture by BPA-treated testes referred to testosterone secreted by the respective contralateral control testes; n = 6 (ERα+/+), 13 (ERα+/−) and 5 (ERα−/−), * p<0.05 in the statistical comparison between BPA-treated and control testes using the Wilcoxon’s parametric paired t test.

We observed that 10^−5^ M BPA reduced testosterone production with testes from ERα−/− as well as with ERα+/− or ERα +/+ fetuses suggesting that ERα is not necessary for BPA action (Fig. 7).

## Discussion

### I - Concentrations of BPA from 10^−8^ M Upwards Affect the Function of Human Fetal Leydig Cells

BPA concentration in biological fluids has been extensively evaluated. BPA is mostly metabolized into BPA glucuronide(s) and BPA sulfate(s) but, unconjugated BPA, which is the only biologically active form [49], has been detected in the majority of individuals in many developed countries with large inter-individual variations. No data on the concentration of BPA found in the plasma of human fetus during the first trimester are presently available. Vandenberg et al. recently reviewed the data from 17 studies published between 2000 and 2009 dealing with the concentration of unconjugated BPA in blood and serum samples from healthy male and non-pregnant female adults. This review indicates that internal exposure to unconjugated BPA is in the range of 2 to 40.10^−9^ M with most studies suggesting an average internal exposure of approximately 4 to 13.10^−9^ M (1 to 3 ng/mL) [50]. BPA concentration in the blood or serum from pregnant women may be slightly higher since different studies reported a mean value of 17.10^−9^ M (3.88 ng/mL) [50]. However, in another study, using isotopically labeled BPA, Teeguarden et al. showed that free BPA represents between 0.2 and 1.2% and no more than 2% of the total BPA in blood, leading to a concentration of free BPA in the plasma lower than 10^−10^ M [51]. Thus, the value of the BPA level of exposure is unclear. Nonetheless, BPA quantification in amniotic fluid and in umbilical cord (to estimate the internal exposure of the fetus) reported a mean level around 1 ng/ml (i.e. 4.3.10^−9^ M) (reviewed in [26]). In a recent study, the concentration of unconjugated BPA was measured in 106 samples of umbilical cord blood in control newborn and concentrations ranged from 0.14 to 4.76 ng/ml (i.e. 0.6 to 20.8.10^−9^ M) with a mean value equal to 1.12 ng/ml (i.e. 4.9.10^−9^ M) [26]. Furthermore, it has been proposed that BPA might accumulate particularly in early fetuses because of lower metabolic clearance or conjugation at this development stage [52]. Taken together, our observation that a concentration of BPA as low as 10^−8^ M (2.28 ng/mL), is able to decrease testosterone secretion and mRNA levels of *INSL3* in human fetal testis can be related to many, but not all reports concerning the internal exposure. Because there is still great uncertainty, and dispute, about the level of effective exposure in humans, it is presently not possible to conclude whether the present findings are health-relevant or not. This will only be resolved once more accurate data for internal human exposure, including the fetus at the first trimester of pregnancy, are established.

The potential dangers for human health of BPA at doses that are below the regulatory references are currently a matter of debates within the industry, the Agencies for Public Health and the scientific community [17], [53]. However, so far, no experimental studies have evaluated the effect of low concentrations of BPA that might correspond to common environmental exposure on the endocrine activities of fetal testis in human and animals, although fetal testis is one of the major target of EDs [6], [7], [54].

Moreover, data dealing with the effect of BPA on the function of human adult Leydig cells are contradictory. Several epidemiological studies reported no changes in testosterone production in adults exposed to BPA, although weak inverse correlations of the testosterone/LH ratio and of estradiol/testosterone ratio with BPA levels were observed [55]–[57]. Conversely, others described a positive association between BPA and plasma testosterone concentrations [58]. Lastly, *in vitro* studies suggested that BPA could decrease steroidogenesis in a human adenocarcinoma cell line or in human microsomal preparations of human testes only at concentrations starting from 1.3 10^−7^ M or 10^−5^ M [45], [59].

### II Comparison with Rat and Mouse

Using the same experimental approach, the same consumables and experimental materials, we also analyzed the effect of the same range of BPA concentrations on the function of Leydig cells in testes from rat and mouse fetuses. Furthermore, the studies of the three species were performed at a comparable developmental stage (*i.e*. the onset of steroidogenesis). No effects of the lowest tested doses (10^−7^, 10^−8^ and 10^−12^ M) were observed as BPA reduced the *in vitro* testosterone secretion only at concentrations equal 10^−5^ M. Similarly, 10^−8^ M BPA did not affect *Insl3* mRNA levels in rat and mouse fetal testes, differently from what we observed in human testes.

These species-specific differences are very important because animal models are routinely used in toxicological tests for the assessment of the BPA risk. Concerns about data extrapolation from *in vivo* animal studies to human risk assessment have already been raised, because BPA metabolism and pharmacokinetics may be different in rodents and in humans [53], [60], [61]. The present study reinforces these concerns by reporting interspecies differences in the responsiveness to BPA and clearly indicates that rodents are not relevant models for predicting the effect of low BPA concentrations on the endocrine function of human fetal testis. This is in agreement with our previous findings showing the human testes are more sensitive to cadmium exposure than rodent testes [40], while MEHP modifies testosterone production in mouse and rat, but not in human fetal testes [36]–[38]. These results highlight the need of rigorous comparisons of the effects in human and rodent models together, when assessing ED risk.

It is difficult to compare the sensitivity to BPA in rodent fetal-type (this study) and in adult-type Leydig cells because most of the investigations in adult animals were performed *in vivo* and BPA can affect the hypothalamo-pituitary-testicular axis at different levels [62]. Cultured adult rat Leydig cells were used only in two studies, but their results are confusing since one work reported that 1 to 1000.10^−9^ M BPA had not effect on the production of testosterone [63], whereas the other observed a negative effect of BPA only at 10^−11^ M, but not at higher doses (10^−10^ to 10^−6^ M) [64].

### III Mechanisms of Action

We observed here that 10^−6^ M and 10^−5^ M DES reduced fetal testicular steroidogenesis in rat and mouse fetal testes confirming our previous data [34], [35], [48]. On the contrary, using the same *in vitro* system, DES had no effect on testosterone produced by fetal human testes. This probably results from the fact that ERα expression (mRNA and protein) is not detected in human fetal testes [65] while DES acts on testicular steroidogenesis via ERα as shown by the disappearance of DES effect after ERα invalidation [48].

BPA binds to nuclear Estrogen Receptors (ERα and ERß), but its affinity for these receptors is weak [66]. This low affinity and the non-detectable expression of ERα in the human fetal testis suggest that ERα is not involved in the effect of BPA in human species. This is probably the same for mouse species since we observed here that the negative effect of BPA on mouse testicular steroidogenesis is maintained after invalidation of ERα.

Therefore, BPA should act via a signaling way different from ERα signaling and this pathway should be more efficient in human than in mouse and rat. Low concentrations of BPA have been reported to trigger effects via G-protein coupled receptor 30 (GPR 30, the membrane estrogen receptor) or Estrogen-related receptor gamma (ERR-gamma) [17], [67], [68]. Both GPR30 and ERR-gamma are expressed in the mouse and human fetal testis (personal observation). Interestingly, ERR-gamma has higher affinity for BPA than for DES [69]. The mechanism of action of BPA may be complex and it has been suggested that BPA might antagonize the binding of an unknown ligand of ERR-gamma that would be constitutively active [69]. More experiments must be conducted to identify the mechanism of action of BPA and the present study gives important methodological bases for conducting these experiments.

### Conclusion

We show here for the first time that concentrations of BPA as low as 10^−8^ M are sufficient to reduce fetal testis endocrine activity in humans. The mechanism of action of BPA will need further investigation, but it is likely to involve non-classical estrogen receptors.

The negative effect of BPA on testosterone production and Insl 3 expression during fetal life observed here during the “masculinization programming window”, may have several consequences as it can impair the masculinization of internal and external genitalia [4], [7], [13]. Our present results should encourage prospective epidemiological studies to investigate the possible association between environmental exposure to BPA during pregnancy and anogenital distance at birth. Furthermore, BPA-induced reduction of fetal testosterone production may have long-term consequences. Although brain masculinization and the onset of male-specific sexual behaviors start during the second half of pregnancy, we can’t exclude that BPA-induced reduction of fetal testosterone production during the first trimester of pregnancy may influence these parameters [70]. It may also affect the germ cell lineage since androgens control gonocyte development [71]. Thus, it will be relevant to study the effect of BPA on gonocytes since alterations of the germ cell lineage may result in testicular cancer or in reduced sperm production [3], [4] and may also explain the recently reported multigenerational effects of BPA [72].

Finally, our findings challenge the widespread use of rodent models to assess the toxic effects of EDs and highlight the need for setting up specific tools to study reprotoxicity in humans.
